# Intracellular Zn(II) Intoxication Leads to Dysregulation of the PerR Regulon Resulting in Heme Toxicity in *Bacillus subtilis*

**DOI:** 10.1371/journal.pgen.1006515

**Published:** 2016-12-09

**Authors:** Pete Chandrangsu, John D. Helmann

**Affiliations:** Department of Microbiology, Cornell University, Ithaca, New York, United States of America; A*STAR, SINGAPORE

## Abstract

Transition metal ions (Zn(II), Cu(II)/(I), Fe(III)/(II), Mn(II)) are essential for life and participate in a wide range of biological functions. Cellular Zn(II) levels must be high enough to ensure that it can perform its essential roles. Yet, since Zn(II) binds to ligands with high avidity, excess Zn(II) can lead to protein mismetallation. The major targets of mismetallation, and the underlying causes of Zn(II) intoxication, are not well understood. Here, we use a forward genetic selection to identify targets of Zn(II) toxicity. In wild-type cells, in which Zn(II) efflux prevents intoxication of the cytoplasm, extracellular Zn(II) inhibits the electron transport chain due to the inactivation of the major aerobic cytochrome oxidase. This toxicity can be ameliorated by depression of an alternate oxidase or by mutations that restrict access of Zn(II) to the cell surface. Conversely, efflux deficient cells are sensitive to low levels of Zn(II) that do not inhibit the respiratory chain. Under these conditions, intracellular Zn(II) accumulates and leads to heme toxicity. Heme accumulation results from dysregulation of the regulon controlled by PerR, a metal-dependent repressor of peroxide stress genes. When metallated with Fe(II) or Mn(II), PerR represses both heme biosynthesis (*hemAXCDBL* operon) and the abundant heme protein catalase (*katA*). Metallation of PerR with Zn(II) disrupts this coordination, resulting in depression of heme biosynthesis but continued repression of catalase. Our results support a model in which excess heme partitions to the membrane and undergoes redox cycling catalyzed by reduced menaquinone thereby resulting in oxidative stress.

## Author Summary

Zinc (Zn(II)) is often considered to be a “first among equals” in metal ion homeostasis. Zn(II) is critically important to the proper function of many cellular processes, yet is toxic at high levels. The molecular basis for Zn(II) intoxication is poorly understood. Using a forward genetic approach in *B*. *subtilis*, we demonstrate that elevated levels of external Zn(II) inhibit the electron transport chain, whereas intracellular Zn(II) intoxication is due to dysregulation of heme biosynthesis. Since the host immune system utilizes both Zn(II) sequestration and toxicity as a means of responding to pathogens, these findings contribute to our understanding of host-microbe interactions.

## Introduction

Approximately 30% of proteins require a metal cofactor. Unlike iron (Fe(II)), which can generate cell damaging hydroxyl radicals in the presence of hydrogen peroxide (Fenton reaction), Zn(II) is not redox reactive. As a result, Zn(II) is favored as a structural cofactor that facilitates folding of a large number of proteins, and is also widely used as a Lewis acid catalyst. Total cellular Zn(II) (the Zn(II) quota) must be high enough to perform these essential roles. Yet, since Zn(II) binds much more avidly to common protein ligands than Fe(II) or Mn(II) (an observation codified in the Irving-Williams series; [1]) excess Zn(II) may result in mismetallation of proteins requiring these other metals. Thus, cellular Zn(II) is highly regulated at multiple levels: in *Bacillus subtilis* the total intracellular concentration at equilibrium is ~0.8 mM, and much of this is sequestered in metalloproteins. A subset of intracellular Zn(II) comprises a labile pool which buffers the thermodynamically free Zn(II) concentration in the picomolar range (< 1 Zn(II) per cell) [2,3], thereby ensuring that only physiologically relevant Zn(II) metalloproteins are normally metallated by Zn(II).

The narrow range of free Zn(II) in *Bacillus subtilis* is set by the transcription repressors Zur, the sensor of Zn(II) limitation, and CzrA, the sensor of Zn(II) excess [4–6]. *B*. *subtilis* contains one high affinity uptake system (*znuABC*) and two efflux systems (*cadA* and *czcD*). Under conditions of Zn(II) sufficiency, Zn(II)-binds to Zur [7], which represses transcription of the Zn(II) uptake systems. Upon Zn(II) deficiency, transcription is depressed and Zn(II) is imported into the cell [4]. When Zn(II) is in excess, CzrA binds Zn(II) and is inactivated [8], leading to depression of CadA and CzcD and Zn(II) efflux [5]. These metalloregulators sense the labile Zn(II) pool consisting of Zn(II) bound to small molecules, proteins and other macromolecules in a kinetically accessible form. In *B*. *subtilis* and related low G+C Firmicutes, the abundant LMW thiol, bacillithiol (BSH), serves as a major buffer of the labile Zn(II) pool [3]. These buffering systems maintain labile Zn(II) concentrations high enough for metallation of Zn(II) containing proteins, but low enough to reduce mismetallation.

The specific targets of zinc intoxication are not well defined. In this study, we take advantage of the well characterized Zn(II) homeostasis mechanisms in the model Gram-positive bacterium, *B*. *subtilis*, and use a forward genetic approach to investigate the underlying causes of Zn(II) intoxication. Our results suggest that in wild type cells, which are competent for export of Zn(II) from the cytosol, Zn(II) intoxication results from inactivation of the electron transport chain due to inhibition of the major aerobic cytochrome *aa*_*3*_ oxidase. Zn(II) resistant suppressors arise that either reduce access of Zn(II) to the cell surface or increase expression of the alternative anaerobic cytochrome *bd* oxidase due to inactivation of Rex, a NAD^+^/NADH sensing transcription factor. Conversely, in a Zn(II) efflux deficient mutant (*cadA czcD*), Zn(II) intoxication results from mismetallation of cytosolic proteins. Here, we identify heme accumulation as a major consequence of intracellular Zn(II) intoxication, which in turn results from mismetallation and consequent dysregulation of the PerR regulon.

## Results

### Isolation of Zn(II) resistant suppressors in wild-type *B*. *subtilis*

To identify potential targets of Zn(II) toxicity and genes involved in Zn(II) resistance, we isolated Zn(II) resistant mutants. A *mariner* transposon library was generated in wild-type cells and plated on a Petri plate containing LB medium and a continuous Zn(II) gradient (0–5 mM). Colonies able to grow in the highest Zn(II) concentrations were isolated and the location of the transposon insertion was identified. We isolated multiple independent transposon insertions in *rex*, *ykuI*, and the *fla-che* operon (Table 1). We backcrossed the transposon insertions into the parental strain by chromosomal DNA transformation. These reconstructed strains, as well as targeted gene deletions, phenocopied the originally isolated Zn(II) resistant transposon mutants, suggesting that the observed Zn(II) resistance is linked to the transposon insertion rather than a second site mutation.

**Table 1:** Isolated Zn(II) resistant suppressors

**Background**	**Gene**	**Total # of unique insertions / mutations**
**WT**	*rex*	4
	*ykuI*	3
	*fla-che* operon	3 in *fliI*, 1 each in *fliF*, *fliK*, *flgD*, *fliQ*, *flhA*, *flhF*
**Background**	**Allele**	**Genotype**
***cadA czcD***	*aroB-1*	insertion at nucleotide 78 (GA)
	*aroB-2*	transversion at nucleotide 35 TCA->TGA (stop)
	*aroB-3*	transversion at nucleotide 79 GAA->TAA (stop)
	*aroB-4*	Δ 143–151
	*aroC-1*	Δ 33–42
	*aroC-2*	Δ 86–88
	*aroC-3*	insertion at nucleotide 207 (A)

YkuI is a c-di-GMP binding protein [9] known to affect production of extracellular matrix (ECM) in *B*. *cereus* [10]. The *fla-che* operon contains genes encoding components of the flagella and chemotaxis machinery, as well as the alternative sigma factor, σ^D^ [11]. ECM production is inversely controlled with respect to flagellar motility in *B*. *subtilis* [12,13]. We therefore hypothesized that the *ykuI* and *fla-che* disruptions prevent Zn(II) intoxication by increasing production of ECM which can prevent access of Zn(II) to the cell, rather than by altering a target of mismetallation. In contrast, Rex is a regulator of anaerobic metabolism and is not known to affect ECM production.

To test whether the *ykuI* and the *fla-che* transposon mutants serve to restrict access of Zn(II) to the cell, we monitored intracellular Zn(II) levels after Zn(II) shock in each of the isolated suppressors (S1 Fig). We reasoned that mutations that restrict access of Zn(II) to the cell, and thereby reduce uptake, would not accumulate Zn(II). Conversely, those that allow the cell to circumvent metabolic pathways intoxicated by Zn(II) would still accumulate Zn(II) upon Zn(II) shock. In contrast with wild-type, strains with transposon insertions in *ykuI* and the *fla-che* operon did not accumulate intracellular Zn(II) upon shock, whereas those with insertions in *rex* did (S1 Fig). These results support the notion that *ykuI* and *fla-che* operon insertions restrict access of Zn(II) to the cell, presumably by increasing ECM production. Since our goal in this study is to define mechanisms of Zn(II) intoxication, we focus here on the role of *rex* in Zn(II) resistance.

### Derepression of *cydAB* is critical for Zn(II) resistance in wild-type *B*. *subtilis*

Rex is a DNA-binding transcription repressor that senses the intracellular NAD/NADH^+^ ratio and regulates genes involved in growth under anaerobic conditions in many Gram positive bacteria such as *S*. *coelicolor* and *B*. *subtilis*, including the cytochrome *bd* terminal oxidase (*cydABCD*), lactate dehydrogenase (*lctP-ldh)*, and a putative nitrate transporter (*ywcJ*) [14–16]. Since a transposon insertion in *rex* leads to increased Zn(II) resistance, we hypothesized that derepression of one or more members of the Rex regulon contributes to Zn(II) resistance.

**Fig 1:**
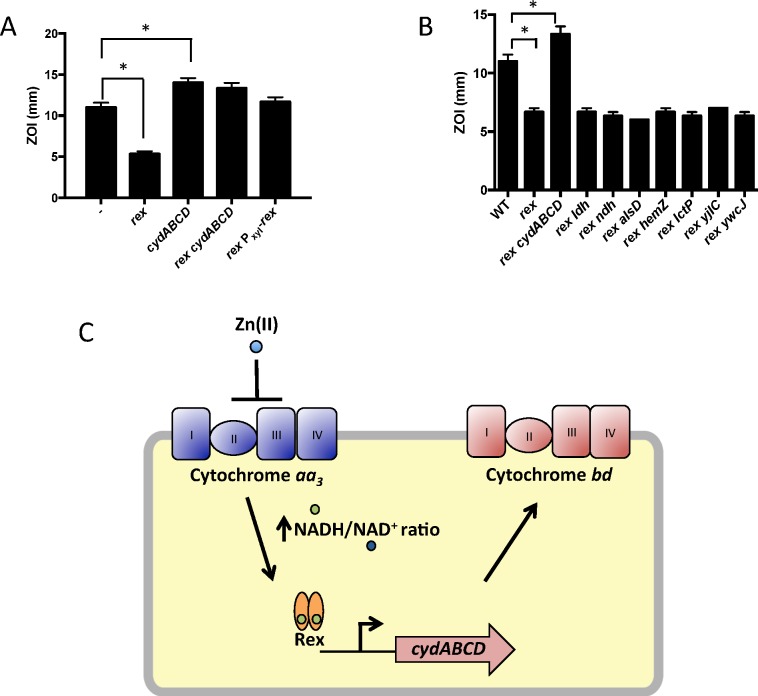
Derepression of the alternative cytochrome *bd* oxidase contributes to Zn(II) resistance. (A and B) Susceptibility of WT and mutant strains to Zn(II) as assessed by disk diffusion assay. The data are expressed as the diameter of the zone of inhibition (ZOI) as measured in millimeters. The mean and standard error of three independent experiments is shown. Asterisks indicate significance as determined by a Student’s *t*-test (P<0.05). (C) Model of the contribution of the Rex regulon to Zn(II) resistance.

**Fig 2:**
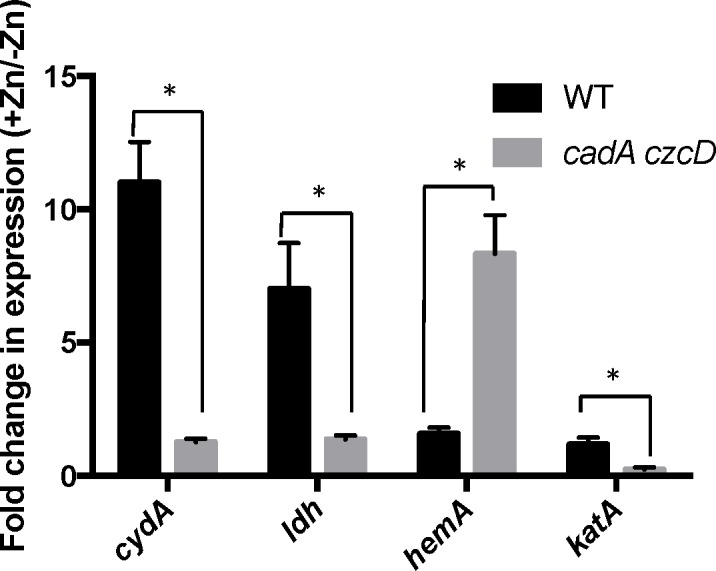
Zn(II) intoxication leads to derepression of the Rex regulon in wild type cells, and dysregulation of PerR in a Zn(II) efflux deficient mutant. Gene expression levels of representative members of the Rex (*cydA* and *ldh*) and PerR regulons (*hemA* and *katA*) was monitored after exposure to Zn(II) (200 μM for WT, 50 μM for *cadA czcD*). Asterisks indicate significance as determined by a Student’s *t*-test (P<0.05).

We constructed mutants in which each Rex-regulated gene was individually deleted in a wild-type or Δ*rex* background. Deletion of *cydABCD* resulted in a Zn(II) sensitive phenotype in a wild-type background (Fig 1A), while there was no Zn(II) phenotype associated with deletion of any other member of the Rex regulon. Additionally, deletion of *cydABCD* in a Δ*rex* background completely reversed the Zn(II) resistance phenotype of the Δ*rex* mutant, consistent with the idea that derepression of *cydABCD* confers Zn(II) resistance (Fig 1B). Furthermore, expression of the Rex regulon is derepressed under conditions of Zn(II) intoxication as measured by qRTPCR of *cydA* and *ldh* expression (Fig 2).

*B*. *subtilis* encodes three terminal oxidases, cytochrome *caa*_*3*_, *aa*_*3*_, and *bd* [17]. Cytochrome *caa*_*3*_ and *aa*_*3*_ are heme-copper oxidases, whereas the relatively less efficient cytochrome *bd* oxidase does not utilize copper. The major cytochrome oxidase used during exponential growth is cytochrome *aa*_*3*_ [18]. Interestingly, expression of either cytochrome *aa*_*3*_ or cytochrome *bd* is required for viability [18]. Thus, during Zn(II) intoxication, the expression of the relatively Zn(II) insensitive cytochrome *bd* terminal oxidase may be required since the major aerobic system, cytochrome *aa*_*3*_, is inhibited by Zn(II) (Fig 1C). This is consistent with prior findings in *Escherichia coli* and *Streptomyces coelicolor* that suggest a similar extracellular target of Zn(II) intoxication [14,19]. Since the ability of Zn(II) to inhibit cytochrome oxidases is well established, we next decided to repeat our selection of Zn(II) resistant mutants using an efflux deficient strain in which Zn(II) intoxication presumably occurs by mismetallation of cytosolic targets.

### Isolation of Zn(II) resistant suppressors in a Zn(II) efflux deficient mutant

We selected spontaneous Zn(II) resistant mutants in a Zn(II) efflux mutant background that lacks the genes encoding the CadA and CzcD Zn(II) efflux pumps. Since the *cadA czcD* mutant displays Zn(II) toxicity at concentrations well below the MIC for wild-type, we reasoned that suppressors isolated from this background would reveal intracellular targets of Zn(II) intoxication. Using whole-genome resequencing, seven independently isolated strains were found to contain nonsense or frameshift mutations in *aroB* and *aroC* (Table 1). No additional mutations were identified in the suppressed strains, suggesting that the Zn(II) resistant phenotype is linked to the inactivation of *aroB* or *aroC*. Consistent with this prediction, strains in which *aroB* or *aroC* were inactivated by insertion of an antibiotic resistant cassette phenocopied the evolved Zn(II) resistant mutants (S2 Fig).

**Fig 3:**
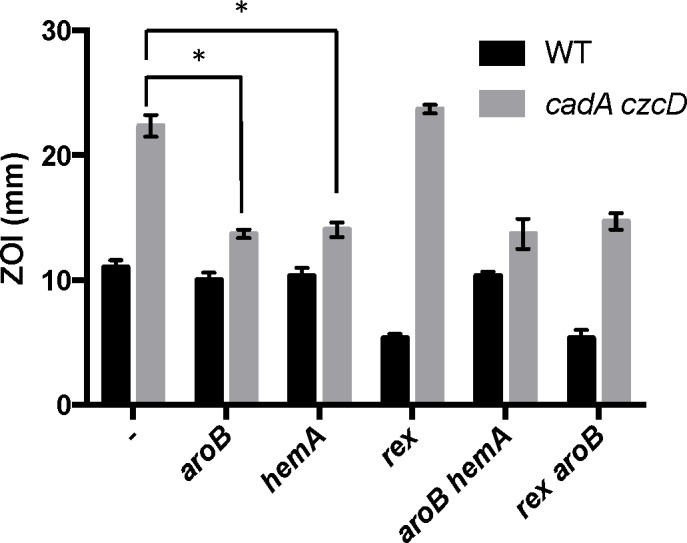
Characterization of Zn(II) resistant suppressors isolated from a Zn(II) efflux deficient mutant. Susceptibility of WT and isolated suppressor strains to Zn(II) as assessed by disk diffusion assay. The data are expressed as the diameter of the zone of inhibition (ZOI) as measured in millimeters. The mean and standard error of three independent experiments is shown. Asterisks indicate significance as determined by a Student’s *t*-test (P<0.05).

Interestingly, *aroB* and *aroC* were not recovered as suppressors of Zn(II) intoxication in the wild-type background (Table 1). This indicates the presence of distinct extracellular and intracellular targets of Zn(II) intoxication. Indeed, each suppressor mutation conferred Zn(II) resistance only in the genetic background in which it was isolated (Fig 3). For example, deletion of *rex* (isolated in wild-type) did not confer Zn(II) resistance to the Zn(II) efflux mutant, and mutation of *aroB* (isolated in the Zn(II) efflux mutant background) did not confer resistance to wild-type (Fig 3).

The *aroB* and *aroC* genes are involved in the biosynthesis of chorismate, a precursor for aromatic amino acid biosynthesis. Since our selection was performed on rich media, it is unlikely that the cells are limited for amino acids. However, chorismate is also a precursor for menaquinone biosynthesis [20]. We therefore hypothesized that Zn(II) resistance may have resulted from a decrease in cellular pools of menaquinone. In *B*. *subtilis*, null mutations affecting late steps in menaquinone biosynthesis fail to form colonies on LB medium [21], whereas the *aroB* and *aroC* null mutants grow well. This suggests that LB medium provides a precursor (perhaps chorismate) that can be used to maintain some level of menaquinone synthesis, and likely accounts for the failure to recover insertions affecting later steps in this pathway.

Transposon insertions in *aroB* and *aroC*, as well as other menaquinone biosynthesis genes, were previously identified in *S*. *aureus* in a selection for heme resistance [22]. The authors suggested a model in which heme toxicity results when superoxide is generated by the redox cycling of membrane-associated heme and reduced quinone molecules [22]. We therefore hypothesized that Zn(II) intoxication in *B*. *subtilis* may also be related to menaquinone and perhaps to heme.

### Zn(II) intoxication requires both menaquinone and heme

**Fig 4:**
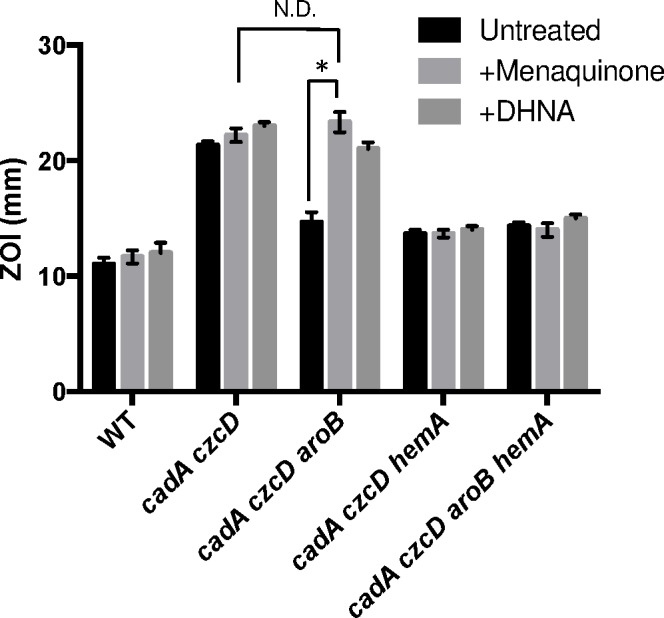
Menaquinone and heme biosynthesis contribute to intracellular Zn(II) intoxication. Susceptibility of WT and mutant strains to Zn(II) as assessed by disk diffusion assay in the presence or absence of 10 μg/ml 1,4-dihydroxy-2-naphthoic acid or menaquinone. The data are expressed as the diameter of the zone of inhibition (ZOI) as measured in millimeters. The mean and standard error of three independent experiments is shown. Asterisks indicate significance as determined by a Student’s *t*-test (P<0.05) and ND indicates no significant difference.

To determine if the relevant effect of the *aroB* and *aroC* mutations is a reduction of menaquinone levels, we measured Zn(II) sensitivity in media supplemented with menaquinone or its precursor 1,4-dihydroxy-2-napthoate (DHNA). We observed that external menaquinone or DHNA supplementation reverses the Zn(II) resistance phenotype of the *cadA czcD aroB* mutant. However, menaquinone or DHNA supplementation does not affect a *cadA czcD hemA* mutant, which is defective in heme biosynthesis (Fig 4). Overall, these data support the hypothesis that an *aroB* mutation leads to Zn(II) resistance by reducing cellular menaquinone levels.

If the role of menaquinone is to act as an electron donor to membrane-localized heme, as proposed for *S*. *aureus* [22], we reasoned that a loss of heme synthesis would also lead to Zn(II) resistance. Indeed, a *cadA czcD hemA* mutant is as Zn(II) resistant as a *cadA czcD aroB* mutant (Fig 4). This strain forms small colonies and is slow growing, thus it not surprising that mutations affecting heme biosynthesis were not isolated in our initial Zn(II) resistance selection. Importantly, the effects of the *aroB* and *hemA* mutations are not additive (compare the *czcD cadA aroB hemA* quadruple mutant with the triple mutants), and this epistasis implies that they act in the same genetic pathway.

### A Zn(II) efflux mutant accumulates heme under conditions of Zn(II) excess

**Fig 5:**
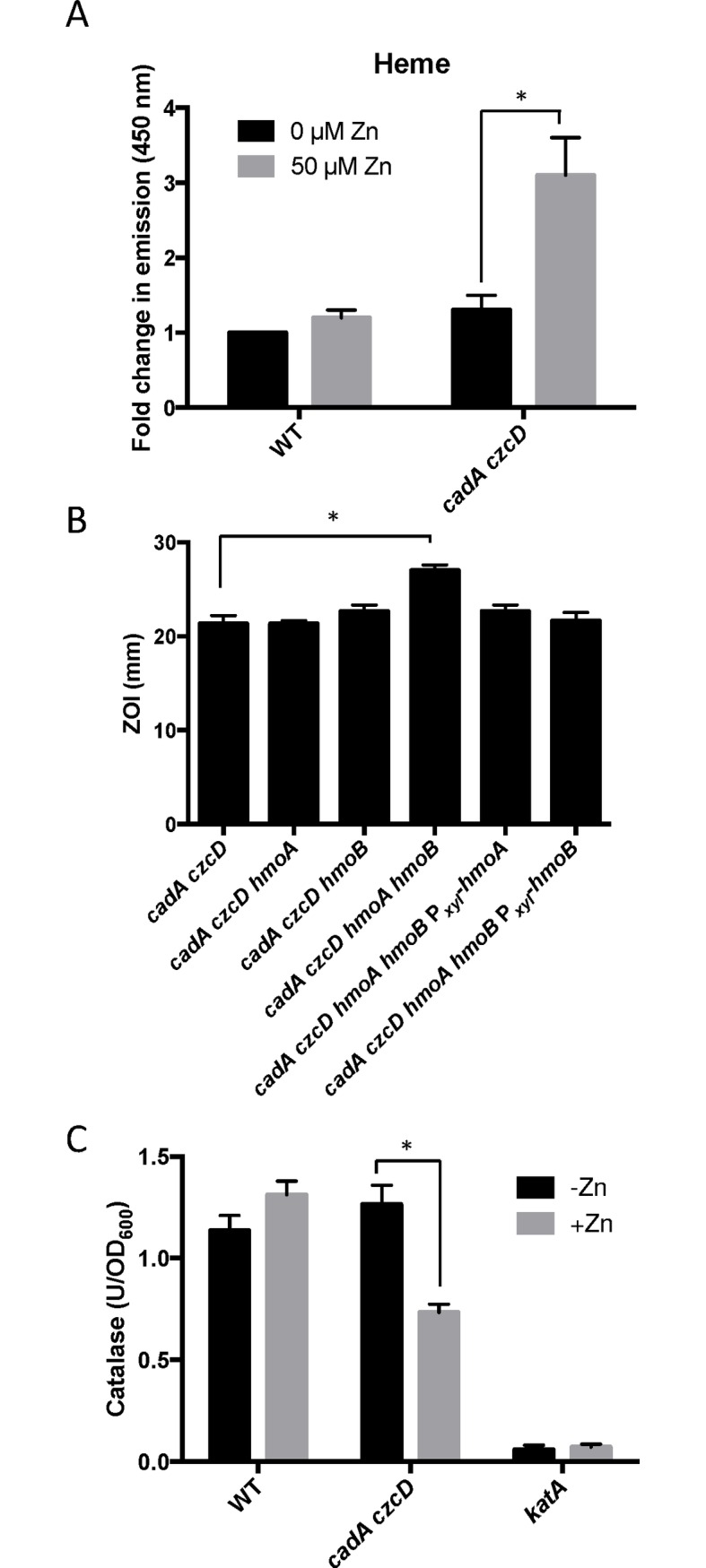
Zn(II) intoxication leads to intracellular heme accumulation. (A) Measurement of heme content of crude extract of cells treated with 50 μM Zn(II). Fluorescence emission from 400 to 500 nm was recorded after excitation at 380 nm. The peak fluorescence intensity at 450 is plotted. The mean and standard error of three independent experiments is shown. (B) Susceptibility of WT and heme monooxygenase mutant strains to Zn(II) as assessed by disk diffusion assay in the presence or absence of external supplementation of menaquinone. The data are expressed as the diameter of the zone of inhibition (ZOI) as measured in millimeters. The mean and standard error of three independent experiments is shown. (C) Catalase activity of wild type and a Zn(II) efflux defective mutant was monitored after a 10 minute exposure to Zn(II) (200 μM for WT, 50 μM for *cadA czcD*). The mean and standard error of three independent experiments is shown. Asterisks indicate significance as determined by a Student’s *t*-test (P<0.05) and ND indicates no significant difference.

To determine if heme accumulates under conditions of Zn(II) intoxication, we used a fluorescence based assay to monitor heme levels. The level of heme increases more than two-fold in the *cadA czcD* mutant background, but not in wild-type (Fig 5A). Although *B*. *subtilis* can utilize heme as an Fe(II) source, it is not known to encode heme uptake or efflux systems. However, *B*. *subtilis* does encode two heme monooxygenases, HmoA and HmoB, that bind and degrade heme in vitro, although a physiological role for these proteins has not yet been demonstrated [23]. HmoB belongs to the well-characterized IsdG family and is not regulated by Fe(II), whereas the Fur-regulated HmoA protein represents a poorly characterized subgroup of monooxygenases found in several pathogenic bacteria [23,24]. Since Zn(II) intoxication leads to heme accumulation, we reasoned that HmoA and HmoB may contribute to Zn(II) tolerance. We tested the Zn(II) sensitivity of *cadA czcD* mutant strains where *hmoA* and *hmoB* were deleted individually or in combination. While deletion of either gene does not have a significant effect, simultaneous deletion of both heme monoxygenases reveals an increased Zn(II) sensitivity in a *cadA czcD* mutant background (Fig 5B). Interestingly, expression of either *hmoA* or *hmoB* from an inducible promoter is able to complement Zn(II) sensitivity of the *cadA czcD hmoA hmoB* mutant. These results suggest that HmoA and HmoB are both active and that they are functionally redundant *in vivo*. Collectively, these data support the hypothesis that intracellular Zn(II) intoxication is due to the toxic redox cycling of excess intracellular heme, as suggested previously in *S*. *aureus* [22]. Next, we sought to find a link between elevated intracellular Zn(II) levels and the observed increase in intracellular heme.

### The PerR regulon is dysregulated under conditions of Zn(II) excess

The heme biosynthesis genes are encoded by the *hemAXCDBL* operon. This operon is repressed by PerR, a metal-cofactored, DNA-binding repressor that serves as a sensor of peroxide stress [25]. In addition, PerR also regulates the expression of catalase (*katA*)[26], the major vegetative catalase in *B*. *subtilis* and an abundant heme binding protein. Coordinate regulation of catalase and heme biosynthesis by PerR ensures sufficient heme availability for catalase function under conditions of oxidative stress.

PerR contains both a structural Zn(II) binding site and a regulatory metal binding site. PerR represses transcription when its regulatory site is associated with either Mn(II) or Fe(II), but only the Fe(II) bound form responds to H_2_O_2_ [27–29]. Since PerR requires a bound regulatory metal ion in order to bind DNA, it also senses conditions of Fe(II) and Mn(II) depletion. *In vitro* data suggest that Zn(II) can also populate the PerR metal sensing site [30], and it may thereby affect the expression of PerR regulated genes. This raises the possibility that under conditions of intracellular Zn(II) intoxication, PerR may become mismetallated with Zn(II), leading to dysregulation of its target genes and to intracellular heme accumulation.

To investigate this hypothesis, we monitored expression of the *hemA* and *katA* genes by qRT-PCR. Upon Zn(II) intoxication, *hemA* mRNA levels increased whereas *katA* mRNA levels decreased in the *cadA czcD* mutant, whereas they were relatively unaffected in a wild-type background (Fig 2). Additionally, catalase activity is also decreased under these same conditions (Fig 5C). This discoordinate regulation contrasts sharply with the documented effects of Fe(II) and Mn(II), which both lead to coordinate regulation of these two operons to allow for sufficient heme production to support catalase activity [31]. These data suggest that the PerR regulon, particularly genes involved in heme biosynthesis and usage, is dysregulated upon Zn(II) intoxication.

**Fig 6:**
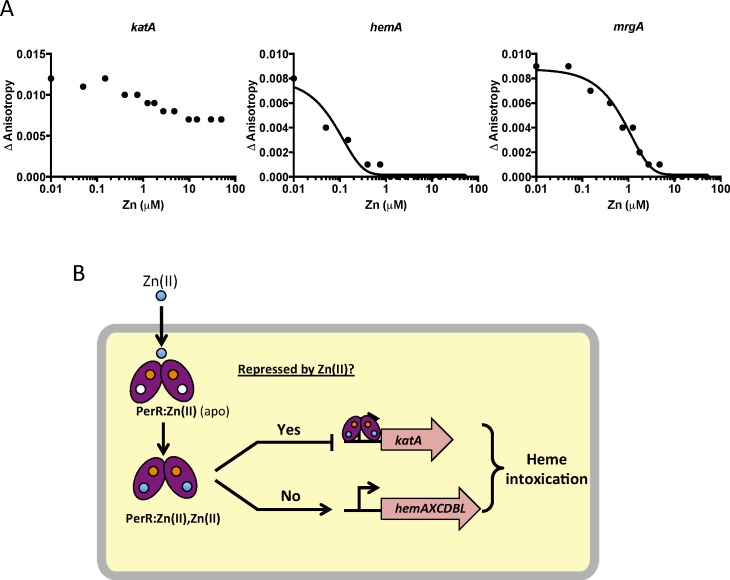
Zn(II) intoxication leads to dysregulation of the PerR regulon. (A) Dissociation of Mn-cofactored PerR (PerR:Zn,Mn) from DNA as monitored by fluorescence anisotropy. Anisotropy was determined with 100 nM DNA containing PerR binding sites from *katA*, *hemA*, and *mrgA* after the addition of 100 nM active PerR dimer and 10 μM MnCl_2_. ZnCl_2_ was titrated at the indicated concentrations. A representative data set is shown. (B) Model for dysregulation of the PerR regulon by Zn(II) intoxication. The structural Zn(II) atom in each monomer is represented by an orange circle and is required for protein folding and stability. The blue circles represent Zn(II) that can enter the cell and, in the absence of efflux, leads to mismetallation of PerR (converting the PerR:Zn,Mn form to a PerR:Zn,Zn form).

To test if this effect is due to direct mismetallation of PerR with Zn(II), we used a fluorescence anisotropy assay to monitor DNA-binding activity. Although PerR binds DNA when bound with either Mn(II) or Fe(II), we routinely use Mn(II) to allow measurement of DNA-binding activity under aerobic conditions [32,33]. As expected, Mn(II)-cofactored PerR binds tightly to 6-carboxyfluorescein-labeled DNA fragments containing binding sites for PerR as judged by an increase in the fluorescence anisotropy signal [34]. Here, we used DNA containing the known PerR operators for the *hemA* operon (encoding heme biosynthesis function), *katA* (encoding the major vegetative catalase), and *mrgA* (encoding a mini-ferritin that sequesters Fe(II) under oxidative stress conditions). The *mrgA* operator is the first identified [35] and the best characterized Per box [36]. Upon titration with Zn(II), a decrease in anisotropy was observed indicative of PerR dissociation from the *hemA* and *mrgA* operator sites (Fig 6A). Interestingly, addition of Zn(II) did not lead to full dissociation of PerR from the *katA* operator site. Collectively, these data suggest that elevated intracellular Zn(II) can lead to the mismetallation of the Fe(II)/Mn(II) sensing transcription factor PerR resulting in a loss of coordination in synthesis of heme and catalase, a major heme-containing protein (Fig 6B).

## Discussion

Metal ion homeostasis relies on the precise control of metal uptake and the complementary action of metal efflux pumps, which prevent intracellular intoxication. Exposure to excess Zn(II), either environmentally or imposed by the innate immune system during infection, can result in growth inhibition [37]. Because of the high efficiency of efflux, Zn(II) often inhibits bacterial growth by binding to extracytoplasmic targets. Two major extracellular targets have been identified previously. In *S*. *pneumoniae*, Zn(II) exerts its toxic effects by preventing the uptake of the essential metal Mn(II) by binding to the Mn(II) solute binding protein, PsaA [38], which delivers Mn(II) to the specific PsaBCD ATP binding cassette (ABC) transporter [39]. Inhibition of PsaA by Zn(II) leaves *S*. *pneumoniae* more sensitive to oxidative stress and more susceptible to killing by the host immune response. Previous studies, and now this work, suggest that a major extracellular target of Zn(II) toxicity in bacteria and mammalian mitochondria is the major aerobic cytochrome oxidase [19,40]. Cytochrome oxidases are key enzymes in aerobic respiration, responsible for establishment of the proton gradient required for ATP synthesis. Measurement of Zn(II) inhibition of the *Rhodobacter sphaeroides* and mitochondrial cytochrome c oxidase indicates an extracellular Zn(II) binding site with K_i_ of 2–5 μM [1,41–43].

We and others have shown that the expression of the alternate anaerobic cytochrome oxidase (*cydBD*) is upregulated under conditions where the major cytochrome oxidase is inhibited by anaerobic conditions [2,14], excess Zn(II) [4,5,14], or excess sulfide [4,44]. Additionally, expression of the *cydBD* cytochrome oxidase is important for survival within a host for *S*. *aureus* [5,45], *M*. *tuberculosis* [46] and *E*. *coli* [47]. Proteomic studies of *S*. *aureus* cells internalized by macrophages reveal an increased level of the anaerobic *cydBD* cytochrome oxidase [48], suggestive of an important role in survival in the host. It is notable that in the case of Zn(II), mutation of *rex* and derepression of the *cydBD* cytochrome oxidase significantly increased Zn(II) tolerance as judged by a zone-of-inhibition assay (Fig 1). Since excess Zn(II) leads to induction of the Rex regulon (Fig 2), we infer that induction of the *cydBD* operon in the presence of Zn(II) does not confer a similar level of Zn(II) tolerance. This suggests that assembly of the CydBD system may be impaired in the presence of excess Zn(II), and what would be an appropriate adaptive response to inhibition of the major aerobic oxidase(s) is, in this case, inadequate.

Bacteria form biofilms and increase the production of extracellular matrix (ECM) in response to a variety of environmental stresses [49]. In both *Xylella fastidiosa* and *E*. *coli* toxic levels of Zn(II) trigger increased ECM production [50,51]. Here, we isolated transposon insertions in *ykuI* and the *fla-che* operon that likely had a similar effect. YkuI is a c-di-GMP binding protein previously implicated in regulation of ECM [9], and mutations affecting flagellar motility have been shown to increase poly-γ-glutamate, a component of the ECM, in *B*. *subtilis* [12,13]. It remains to be seen if biofilm formation and increased ECM synthesis is be a normal physiological response to Zn(II) stress in *B*. *subtilis*.

The importance of intracellular Zn(II) homeostasis suggests the presence of an intracellular target for Zn(II) toxicity. In Group A *Streptococcus*, key glycolytic enzymes are mismetallated by Zn(II) and production of capsule polysaccharides is inhibited [52]. In *E*. *coli*, intracellular Zn(II) toxicity under conditions of oxidative stress results from mismetallation of the iron sulfur clusters of dehydratases, which are critical to key metabolic processes [53,54].

We sought to identify additional proteins that could be mismetallated by Zn(II) and the underlying mechanisms of Zn(II) intoxication in *B*. *subtilis*. By isolation of suppressors of zinc toxicity in a Zn(II) efflux mutant, we identified PerR as a target of mismetallation by Zn(II). PerR has been implicated in Zn(II) homeostasis in *S*. *pneumoniae* since it regulates expression of the P_1B4_-type ATPase encoded by *pmtA* [55]. In a *perR* null mutant, overexpression of PmtA leads to induction of a Zn(II) starvation response, implying that this transporter may export Zn(II). However, PmtA is not known to be induced by Zn(II) stress, and its physiological role is most likely related to peroxide resistance (as evidenced by its regulation by PerR). Indeed, recent studies of PmtA orthologs in *B*. *subtilis* [56], *L*. *monocytogenes* [57], and *M*. *tuberculosis* [58] (PfeT, FrvA, and CtpD, respectively) suggest that the primary substrate of these P_1B4_-type ATPases is Fe(II), and this is also a likely role for PmtA consistent with the key role this protein plays in peroxide resistance [55].

It is presently unclear why Zn(II) inhibits binding of PerR to some operons, but not others. Prior work suggests that many Fur family proteins may utilize the cooperative binding to effect repression [32,59]. For example, *Corynebacterium diptheriae* DtxR can bind DNA cooperatively as a tetramer [60], and a similar model has been proposed for *E*. *coli* Zur [61]. We note that Zn(II) leads to complete dissociation of PerR from the *hemA* operon regulatory site, but not from *katA* (Fig 6). The regulatory region of *hemA* contains multiple PerR binding sites [28], whereas the *katA* regulatory region includes one very strong consensus site [26]. Therefore, we can speculate that perhaps Zn(II)-metallated PerR is defective in cooperative binding, but can still bind strong consensus sites as a dimer. Regardless of the molecular details, Zn(II) clearly leads to dysregulation of the PerR regulon and an increase in intracellular heme levels. Our work supports the model of heme toxicity proposed by the Skaar lab which suggests that heme toxicity is caused by the generation of superoxide as membrane associated heme and reduced quinones form a redox cycle [22].

Previous studies support the idea that mismetallation of metalloregulatory proteins can have dire consequences. The Fe(II) sensing transcription factor Fur is known to be mismetallated by Mn(II) under Mn(II) intoxication conditions or when Fur levels increase [62]. Recently, Cd(II) intoxication in *S*. *pneumoniae* was linked to dysregulation of Zn(II) homeostasis, resulting from inhibition of Zn(II) uptake gene expression and activation of Zn(II) efflux gene expression [63]. However, direct interaction of Cd(II) with the Zn(II) sensing transcription factor AdcR and SczA has not yet been shown. Additionally, as a shown in Group A *Streptococcus*, under conditions of Mn(II) intoxication, the Fe(II)/Mn(II) sensing transcription factor PerR is in its Mn(II)-cofactored form [64]. As a result, the PerR regulon is unable to be induced by H_2_0_2_, resulting in increased sensitivity to oxidative stress. Similarly, excess Mn(II) blocks catalase de-repression and prevents the increase in H_2_O_2_ tolerance that is typical of the entry of *B*. *subtilis* cells into stationary phase [26].

Understanding the mechanisms of Zn(II) intoxication may contribute to the development of novel antibacterial treatments. Only recently has the role of host-mediated Zn(II) toxicity as an antimicrobial mechanism been widely appreciated: regulated Zn(II) trafficking to the phagosome appears to play an active role in antimicrobial responses in several systems. Phagosomal Zn(II) levels rise dramatically upon *Mycobacterium tuberculosis* [65] or *Streptococcus pyogenes* [66] infection, and Zn(II) containing vesicles have been observed in macrophages upon *Salmonella enterica* Typhimurium infection [67]. Furthermore, many pathogenic bacteria require Zn(II) efflux pumps to avoid killing by macrophages [65,66,68]. Such evidence suggests that high levels of Zn(II) may exert a direct bactericidal effect within macrophages.

## Methods

### Bacterial strains, plasmids, and growth conditions

Strains used in this study are listed in S1 Table. Bacteria were grown in the media described in the following sections. When necessary, antibiotics were used at the following concentrations: chloramphenicol (10 μg ml^-1^), kanamycin (15 μg ml^-1^), spectinomycin (100 μg ml^-1^), and tetracycline (5 μg ml^-1^). Gene deletions were constructed using long flanking homology PCR as previously described [69]. Chromosomal DNA transformation was performed as described.

### Disk diffusion assays

Strains were grown in LB at 37°C with vigorous shaking to an OD_600_~0.4. A 100 μl aliquot of these cultures was added to 4 ml of LB soft agar (0.7% agar) and poured on to prewarmed LB agar plates. The plates were then allowed to solidify for 10 minutes at room temperature in a laminar flow hood. Filter disks (6 mm) were placed on top of the agar and 10 μl of Zn(II) (50 mM) was added to the disks and allowed to absorb for 10 minutes. The plates were then incubated at 37°C for 16–18 hours. The diameter of the zone of inhibition was measured. The data shown represent the values (diameter of the zone of inhibition minus diameter of the filter disk) and standard deviation of three biological replicates.

### Quantification of total Zn(II) quota by ICP-MS

For quantification of the total Zn(II) quota, cells were grown in 5 ml LB medium in the presence or absence of 200 μM ZnCl_2_ to mid-log phase. For fractionation experiments, 25 ml of cells were grown in presence or absence of 200 μM Zn(II). All samples were prepared as described previously. Briefly, samples were washed once with buffer 1 (1X PBS buffer, 0.1 M EDTA) then twice with buffer 2 (1X chelex-treated PBS buffer). Cell pellets were resuspended in 400 μl buffer 3 (1X chelex-treated PBS buffer, 75 mM NaN3, 1% Triton X-100) and incubated at 37°C for 90 min for cell lysis. Lysed samples were centrifuged and subject to Bradford assay to quantify the total protein content. Then, samples were mixed with 600 μl buffer 4 (5% HNO3, 0.1% (v/v) Triton X-100) and heated in a 95°C sand bath for 30 min. Samples were centrifuged and supernatants were diluted in 1% HNO3. Levels of intracellular Zn were analyzed by Perkin-Elmer ELAN DRC II ICP-MS. Gallium was used as an internal standard. The total concentration of metal ions is expressed as μg ion per gram of protein. The data shown represent the average and standard deviation of three biological replicates.

### qRT-PCR

Cells were grown at 37°C in LB medium with rigorous shaking till OD_600_ ~0.4. 1 ml aliquots were treated with ZnCl for 10 min. Total RNA from both treated and untreated samples were extracted RNeasy Mini Kit following the manufacturer's instructions (Qiagen Sciences, Germantown, MD). RNA samples were then treated with Turbo-DNA free DNase (Ambion) and precipitated with ethanol overnight. RNA samples were re-dissolved in RNase-free water and quantified by NanoDrop spectrophotometer. 2 μg total RNA from each sample was used for cDNA synthesis with TaqMan reverse transcription reagents (Applied Biosystems). qPCR was then carried out using iQ SYBR green supermix in an Applied Biosystems 7300 Real Time PCR System. 23S rRNA was used as an internal control and fold-changes between treated and untreated samples were plotted.

### Catalase activity

Cells were grown on LB agar plates overnight. A colony was picked from the fresh plate and placed in a drop of dilute hydrogen peroxide. Strains resulting in the formation of bubbles were determined to possess catalase activity. For quantitative determinations of catalase activity, cells were assayed for the rate of H_2_O_2_ decomposition spectrophotometrically (240 nm) as described previously [70].

### Overexpression and purification of PerR

pET16b plasmids carrying PerR between the NcoI and BamHI restriction sites were used for overexpression in *Escherichia coli*. A single colony was inoculated into 10 ml of LB and grown overnight at 37°C. Cells were then diluted into 1 L of LB medium containing 0.5% glucose. 1 mM IPTG was added to the culture when OD600 reached ∼0.6 and cells were harvested after 2 h of induction by centrifugation. After resuspension and sonication in buffer A [20 mM Tris, pH 8.0, 100 mM NaCl, 5% (v/v) glycerol] with 10 mM EDTA (ethylenediaminetetraacetic acid), the supernatant were loaded onto a heparin column pre-equilibrated with the same buffer. Proteins were eluted with a linear gradient of NaCl from 0.1 to 1 M and fractions containing PerR were combined and concentrated to load onto a Superdex 200 size exclusion column. The recovered PerR eluted was further purified using a Mono-Q column with a linear gradient of 0.1–1 M NaCl in buffer A containing 10 mM EDTA. Fractions containing PerR were concentrated and dialyzed extensively against buffer A to remove EDTA. The purified PerR was then aliquoted and frozen at −80°C. The purified PerR was determined to be ∼85% active, respectively, based on the amount of protein required to stoichiometrically bind 100 nM DNA.

### Fluorescence anisotropy

A 6-carboxyfluorescein (6-FAM)—labeled DNA was used and fluorescence anisotropy (FA) was measured with λ_ex_ = 492 nm and λ_em_ = 520 nm. For Zn(II) binding, increasing amounts of ZnCl_2_ were mixed with 120μl buffer A containing 100 nM labeled DNA, 100 nM active PerR dimer, and 10 μM MnCl_2_. FA measurements of each sample were performed immediately after transferring to a quartz cuvette. The data were fit using a nonlinear regression model with GraphPad Prism.

### Fluorescence-based PPIX and heme assays

Strains were grown overnight in LB medium and then subcultured with 1:100 ratio in fresh LB medium to an OD600 of 0.4. OD600 was recorded and aliquots of 5 ml of cell culture were harvested. Cell pellets were resuspended in 1 ml 50 mM Tris-HCl buffer (pH 7.4) containing 100 μg ml−1 of lysozyme and were incubated at 37°C for 30 min to lyse the cells. Cell debris was removed by centrifugation. Heme was extracted from 500 μl of the clear lysates using 500 μl acidic acetone (20% (v/v) 1.6 M HCl). Precipitate was removed by centrifugation and the supernatant was analyzed by fluorescence spectroscopy. The fluorescence emission of heme was scanned from 400 nm to 500 nm with excitation at 380 nm as described. Fluorescence intensity at 450 nm (peak) was normalized and plotted.

## Supporting Information

S1 Fig Intracellular Zn(II) concentration of isolated Zn(II) resistant suppressor mutants after Zn(II) shock. ICP-MS was used to measure the intracellular Zn(II) concentration for (A) wild-type and (B) *cadA czcD* mutant after exposure to 200 μM (for WT) and 50 μM (for *cadA czcD*) ZnCl_2_ at indicated time points. The mean and standard error of three independent experiments is shown.
(TIF)

S2 Fig Characterization of spontaneously occurring Zn(II) resistant suppressors in a Zn(II) efflux (*cadA czcD*) deficient mutant. Susceptibility of wild-type, *cadA czcD*, and isolated suppressor strains to Zn(II) as assessed by disk diffusion assay. The data are expressed as the diameter of the zone of inhibition (ZOI) as measured in millimeters. The mean and standard error of three independent experiments is shown. Asterisks indicate significance as determined by a Student’s *t*-test (P<0.05).
(TIF)

S1 Table Strains used in this study. (DOCX)
