# Supplementary figures and images for: Intracellular Zn(II) Intoxication Leads to Dysregulation of the PerR Regulon Resulting in Heme Toxicity in Bacillus subtilis
Source: PLoS Genet. 2016 Dec 9;12(12):e1006515. doi: 10.1371/journal.pgen.1006515 (PMC5189952; doi:10.1371/journal.pgen.1006515)

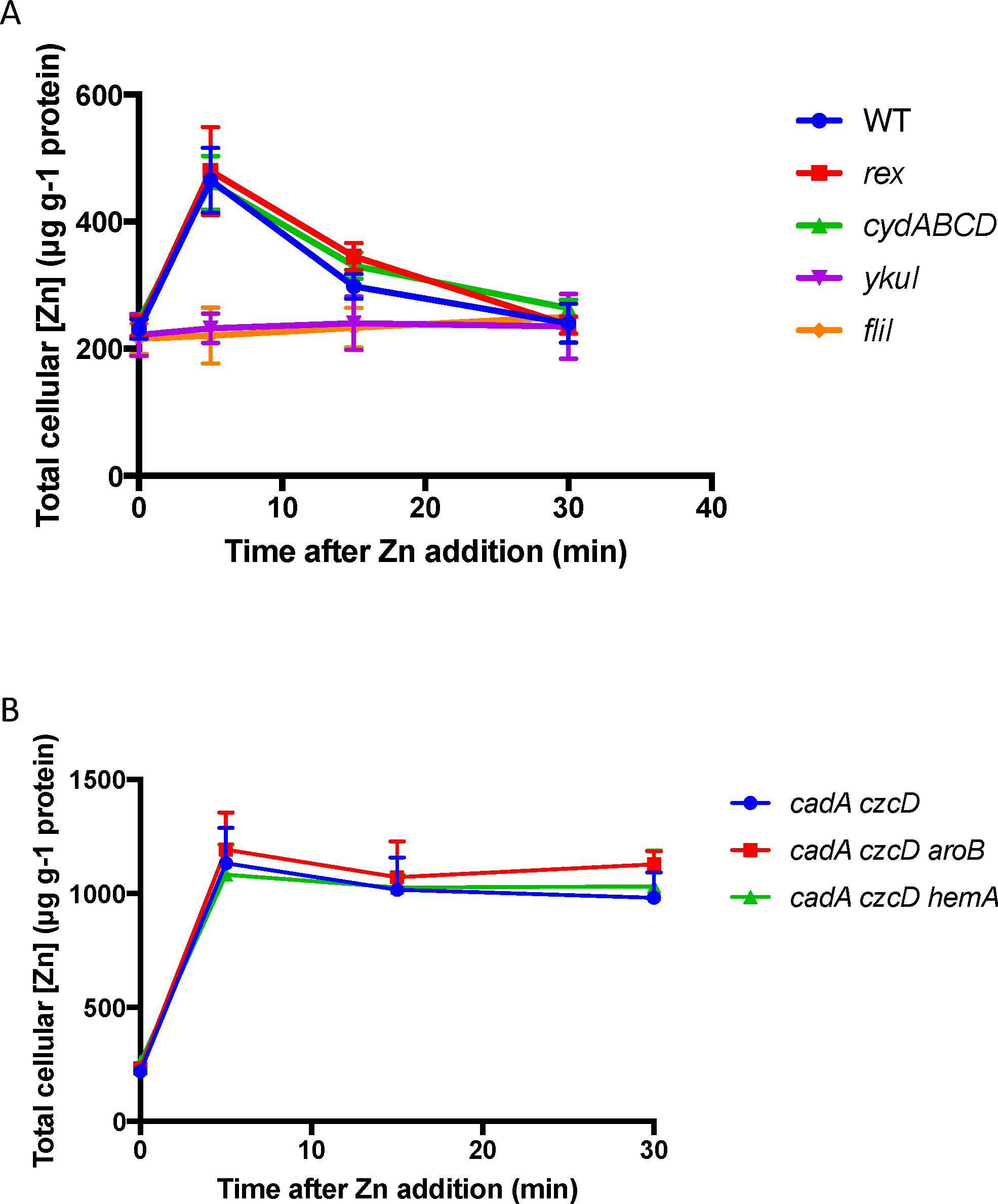

Supplement: S1 Fig — ICP-MS was used to measure the intracellular Zn(II) concentration for (A) wild-type and (B) cadA czcD mutant after exposure to 200 μM (for WT) and 50 μM (for cadA czcD) ZnCl2 at indicated time points. The mean and standard error of three independent experiments is shown. (TIF) [file pgen.1006515.s001.tif]

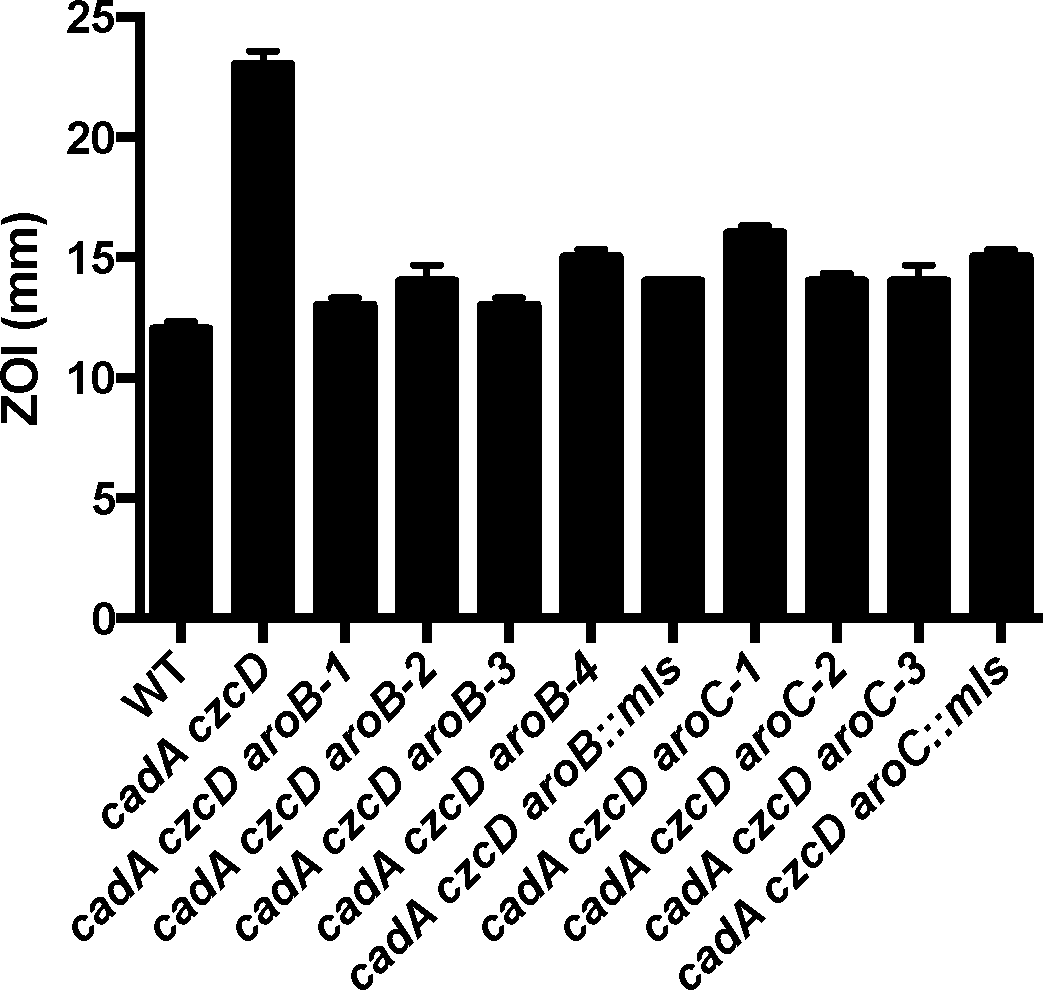

Supplement: S2 Fig — Susceptibility of wild-type, cadA czcD, and isolated suppressor strains to Zn(II) as assessed by disk diffusion assay. The data are expressed as the diameter of the zone of inhibition (ZOI) as measured in millimeters. The mean and standard error of three independent experiments is shown. Asterisks indicate significance as determined by a Student’s t-test (P<0.05). (TIF) [file pgen.1006515.s002.tif]
