# Supplementary material for: Intracellular Zn(II) Intoxication Leads to Dysregulation of the PerR Regulon Resulting in Heme Toxicity in Bacillus subtilis
Source: PLoS Genet. 2016 Dec 9;12(12):e1006515. doi: 10.1371/journal.pgen.1006515 (PMC5189952; doi:10.1371/journal.pgen.1006515)
Supplement: S1 Table — (DOCX) [file pgen.1006515.s003.docx]

| ­­­Strain | Genotype | Reference |
| --- | --- | --- |
| CU1065 | W168 *att* SPβ *trpC2* (WT) | Lab stock |
| HB16586 | CU1065 *rex::spc* | This work |
| HB16587 | CU1065 *cydABCD::mls* | This work |
| HB16588 | HB16586 *cydABCD* | This work |
| HB16589 | HB16586 *amyE::*P_xyl_-*rex* | This work |
| HB16590 | HB16586 *ldh::mls* | This work |
| HB16591 | HB16586 *ndh::mls* | This work |
| HB16592 | HB16586 *alsD::mls* | This work |
| HB16593 | HB16586 *hemZ::mls* | This work |
| HB16594 | HB16586 *lctP::mls* | This work |
| HB16595 | HB16586 *yjlC::mls* | This work |
| HB16596 | HB16586 *ywcJ::mls* | This work |
| HB16597 | CU1065 *aroB::mls* | This work |
| HB16598 | CU1065 *hemA::cm* | This work |
| HB11395 | CU1065 *cadA::kan czcD::tet* | [5] |
| HB16599 | HB11395 *aroB::mls* | This work |
| HB16600 | HB11395 *hemA::cm* | This work |
| HB19401 | HB16599 *hemA::cm* | This work |
| HB19402 | HB11395 *rex::spc* | This work |
| HB19403 | HB11395 *hmoA::spc* | This work |
| HB19405 | HB11395 *hmoB::mls* | This work |
| HB19406 | HB19405 *hmoB::mls* | This work |
| HB19407 | HB19406 *amyE*::P*_xyl_*-*hmoA* | This work |
| HB19408 | HB19406 *amyE::*P_xyl_-*hmoB* | This work |
| HB19409 | CU1065 *katA::mls* | This work |
| ­HB19410 | CU1065 *ykuI::mls* | This work |
